# Validation of a generic impact survey for use by health library services indicates the reliability of the questionnaire

**DOI:** 10.1111/hir.12427

**Published:** 2022-03-25

**Authors:** Christine Urquhart, Alison Brettle

**Affiliations:** ^1^ Aberystwyth University Aberystwyth UK; ^2^ School of Health and Society University of Salford Salford UK

**Keywords:** evaluation, impact, libraries, health care, libraries, medical, questionnaires, research methodology, statistics, surveys, United Kingdom (UK)

## Abstract

**Background:**

A validated generic impact questionnaire can demonstrate how individual and groups of health libraries contribute to continuing education and patient care outcomes.

**Objectives:**

To validate an existing generic questionnaire for Knowledge for Healthcare, England by examining: (1) internal reliability; (2) content validity; and (3) suggest revisions.

**Methods:**

Methods used included Cronbach's alpha test, simple data mining of patterns among a data set of 187 questionnaire responses and checking respondents' interpretation of questions.

**Results:**

Cronbach's alpha was 0.776 (acceptable internal reliability). The patterns of responses indicated that respondents' interpretations of the questions were highly plausible, and consistent. The meaning of ‘research’ varied among different occupational groups, but overall, respondents could identify relevant personal and service impacts. However, users were confused about the terms that libraries use to describe some services.

**Discussion:**

The analysis indicated that the questionnaire worked well for the two types of personal services (literature/evidence searches and training/e‐learning) frequently cited on the responses. Further research may be required for library assessment of the impact of other services such as digital resource services.

**Conclusions:**

The generic questionnaire is a reliable way of assessing the impact of health library and knowledge services, both individually and collectively.

**Key Messages**

- A validated, generic questionnaire to assess the impact of health library and knowledge services in England may be applicable to services elsewhere.
- Validation showed the importance of checking how library service users interpret both impact questions and descriptions of library services.
- An evidence‐based approach to value and impact assessment also demonstrates the importance of collaborative research and development.

## BACKGROUND

This article explains a robust validation of a theoretically grounded, simple questionnaire to routinely measure the impact of the health library contribution on the range of activities performed by health care organisations. Demonstrating impact of health libraries is important to highlight the key role that health libraries and librarians play in supporting health care organisations to achieve their objectives of providing high‐quality, evidence‐based and cost‐effective care. Cost pressures on health care following the COVID‐19 pandemic make provision of that evidence of impact essential for health libraries.

Challenges involved in measuring the impact of libraries are well documented, long‐standing and not restricted to health (Markless & Streatfield, 2012; Matthews, 2015; Oakleaf, 2010; Saracevic & Kantor, 1997; Urquhart & Hepworth, 1995). Measuring impact involves measuring the difference or change in an individual or group resulting from the contact with library services (International Organization for Standardization, 2014). However, these changes may not have immediate, tangible or direct outcomes which can easily be measured. For example, a health library can only make a contribution to patient care, it is unlikely to be able to change or influence patient care without a health professional making the clinical decision. A further issue is that of separating the value of the information obtained from the library, from the value of the library service itself (Urquhart & Turner, 2016).

The Rochester study on the impact of medical library services (Marshall, 1995) and the UK adaptation (Urquhart & Hepworth, 1995) aimed for questionnaire items that were based on previous evidence on the purposes of information use and outcomes relevant to clinical decision making. Evaluation methods and measures should be valid and reliable (Brettle, 2007) to ensure the credibility of the results. Later work aimed to widen impact evaluations across a wider range of staff groups among UK health libraries (Weightman et al., 2009) and later discussions highlighted the need for a shorter questionnaire. Quality assurance data collected annually suggested that existing guidance was not well implemented (Metrics Task and Finish Group, 2016) with libraries developing local, non‐validated, tools and failing to act on the evidence collected (Ayre et al., 2018).

In England, libraries within the NHS are known as Library and Knowledge Services (LKS) and operate within a national strategy and framework (Knowledge for Healthcare, 2015–2020). To demonstrate the contribution made by LKS services, an Evaluation Framework sets out key indicators which libraries should work toward to demonstrate achievement of their success (https://kfh.libraryservices.nhs.uk/ef-intro/). Alongside, LKS are encouraged to use a suite of tools to demonstrate the impact of their services (https://kfh.libraryservices.nhs.uk/value-and-impact-toolkit/) (Edwards & Gilroy, 2021). The tools comprise a generic questionnaire, an interview schedule and a case study template to encourage consistent reporting of “stories” of impact, useful for advocacy purposes. The generic questionnaire is theoretically derived, based on international standards with practitioner input, demonstrates initial validity (face and initial construct) as well as acceptability and feasibility for routine use (Ayre et al., 2018). Fundamental components in establishing the trustworthiness of an instrument are validity (is the test measuring what is intended) and reliability (is the test measuring in a reproducible way) (Streiner & Norman, 2003).

This paper describes further validation of the generic questionnaire providing evidence of its potential usefulness in robustly measuring the impact of health library services. The study objectives were to:

- investigate the internal reliability (quantitative methods);
- content validity (qualitative study);
- present a revised version of the questionnaire for routine use.

## METHODS

We used quantitative and qualitative methods. The quantitative research focused on internal reliability investigations (Cronbach's alpha to assess internal consistency and data mining of the patterns of responses) within a set of generic questionnaire responses (mostly involving the literature/evidence search and the training/e‐learning library services). Using respondents from a library which had not participated in the quantitative data collection, the qualitative research focused on content validity, by interviewing a range of types of respondents with further transcription and analysis of responses to check respondents' interpretation and understanding of the questions. Ethical approval was obtained from the University of Salford Ethics Committee (HSR1920‐044) for the qualitative component.

### Internal consistency using the Cronbach's alpha test

Internal consistency checks whether the items are measuring different aspects of the same underlying dimension (Streiner & Norman, 2003). There are various ways of checking the internal reliability or consistency of a questionnaire, most commonly, Cronbach's alpha. The value depends on how people vary on individual items, relative to how much they vary overall on the test. A low value indicates a wide variation and the value for alpha is low. For this questionnaire (Appendix 1), there will be some variation, as there are different professional groups, different reasons for the using the library services, different uses and different impacts. We used a test set of 187 completed anonymised questionnaire responses collected from various sites and collated by Knowledge for Health senior staff on an Excel spreadsheet. This was then transferred into the SPSS statistical package to perform the Cronbach's alpha test. We attached each library service use as a separate variable on the data so that the SPSS file maintained the individuality of the observation.

We conducted item analysis to explore whether any items should be removed from the questionnaire. This analysis focused on three of the question sets in the questionnaire, focusing on “have used” responses only for:

- Question set 2 on uses of information: ‘From that single use of library services or resources how did you use, or how might you use, the information, knowledge or skills gained? (Tick any that apply)’.
- Question set 3 on personal impacts: ‘From that single use of library services or resources how did the information, knowledge or skills gained help? (Tick any that apply)’.
- Question set 4 on service impacts: ‘Did your use of library resources or services contribute to any of the following impacts? (Tick any that apply).

A high alpha (0.75–1.0) indicates a reliable test. We calculated both the overall alpha (28 items) and the item‐by‐item value (calculating the alpha if that item was removed).

### Internal reliability – Data mining of patterns in responses

A grid table was prepared to collate the responses of the respondents against each question, with category variables used (0, 1, 2) to represent (for example), no response, ‘probably will use’ (information retrieved) or ‘have used’ (information retrieved). For example, for Question 2 (Appendix 1, questionnaire) the data was split into 13 attributes (for each sub‐question) and options coded for each possible response. Data could be partitioned, using conditional statements (‘if and else’, or ‘where’) to find frequencies of response for the specific combination of attributes/coded categories.

We used these simple data mining techniques with the SPSS data set:

- To check whether there were serious discrepancies in the way some of the main occupational groups among the respondents appeared to interpret the questionnaire wording.
- To examine patterns of responses across two or three of the questions, to check, for example, whether a particular use was associated with the type of impacts that might be expected.
- To check the plausibility of the patterns of responses (e.g. in immediate and future contributions to service impact).

Most of the data mining involved working with subsets of the responses and checking the patterns of responses. The Pearson correlation coefficient was used with responses indicating gaining or updating skills, to check that these were scaling satisfactorily.

### Content validity

Content validity helps to establish whether the scale looks reasonable in attempting to measure what it intends to measure; and along with establishing face validity is a minimum pre‐requisite for acceptance of a measure (Streiner & Norman, 2003). Checking the elements in a measure with users of the instrument are important components of establishing content validity (Haynes et al., 1995). We used qualitative interviews to determine content validity, having already established face validity with the librarians (Ayre et al., 2018). Recent library users from a hospital which had not previously distributed the questionnaire were purposively selected and interviewed by the hospital librarian. The librarian arranged the interviews and took informed consent from 10 participants representing each staff group listed on the questionnaire. Following a training session with one of the project team, the librarian interviewed each participant, in person, by taking the participant through the questionnaire and checking understanding of each item. We audio recorded interviews and kept a manual record by marking a copy of the questionnaire to indicate the items needing clarification. We analysed the responses by one project team member listening to each interview and correlating this with the manual records.

## RESULTS

### Internal consistency – Cronbach's alpha

Item analysis (Table 1) indicated that all items performed satisfactorily, with item alpha for the 28‐item list as 0.776 Cronbach alpha value, above the acceptable value of internal consistency (Tavakol & Dennick, 2011).

**TABLE 1:** Item analysis (Cronbach alpha – if item deleted)

Question item	Cronbach alpha if item deleted	Question number
Personal or professional development	0.767	Question 2 (how information, knowledge, or skills gained were used)
Direct patient care	0.750	Question 2
Teaching or presentations	0.756	Question 2
Sharing information with, or advising, other staff or colleagues	0.758	Question 2
Patient information, advising or educating patients, clients or families	0.753	Question 2
Developing guidelines/guidance/pathways/policies	0.756	Question 2
Audit	0.756	Question 2
Research	0.783	Question 2
Organisational/service development/business planning	0.755	Question 2
Legal or ethical questions	0.763	Question 2
Commissioning or contracting	0.761	Question 2
Publication	0.761	Question 2
None of the above	0.777	Question 2
Confirm prior knowledge or refresh my memory	0.783	Question 3 (personal impact)
Gain new knowledge	0.780	Question 3
Generate new ideas	0.784	Question 3
Update skills	0.784	Question 3
Gain new skills	0.784	Question 3
Improve my confidence	0.785	Question 3
Save my time	0.784	Question 3
None of the above	0.762	Question 3
Reduced risk or improved safety	0.758	Question 4 (service impact)
Improved the quality of patient care	0.769	Question 4
Saved money or contributed to financial effectiveness	0.761	Question 4
More informed decision making	0.766	Question 4
Contributed to service development or delivery	0.766	Question 4
Facilitated collaborative working	0.781	Question 4
Contributed to personal or professional development	0.777	Question 4
None of the above	0.767	Question 4

Question 1 (type of library service used) and Question 5 (type of role) were omitted from the Cronbach analysis as they did not concern uses or impacts resulting from a particular instance of library service usage. For the internal consistency of Question 2 (uses of information and Question 4 (service impact, the Cronbach alpha value was 0.839, indicating high reliability. For the internal consistency of Question 2 (uses of information and Question 3 (personal impact, the Cronbach alpha value was 0.713, which is below the normal cut‐off of 0.75 but satisfactory for the type of questions being asked. For any of the items in Question 3, item deletion would improve the reliability of the questionnaire – but it would not improve the usefulness of the questionnaire. The linkages between Question 2 and Question 4 appear more obvious (e.g. ‘patient information’, and ‘improved the quality of patient care’) whereas the personal impacts (Question 3) will vary considerably. They are very dependent on individual knowledge, learning, and experience. And uses of information (Question 2) will also depend on individual circumstances at the time.

### Data mining – Patterns of responses for occupational group and main use

The largest groups of respondents were allied health professionals (*n* = 48), nursing and midwifery (*n* = 47), and medicine and dental (*n* = 45). The most popular use (Question 2) for these groups was personal/professional development (over two thirds in each group). There were considerably fewer respondents from other roles, but personal or professional development was also an important use for those in additional clinical services (*n* = 7), administrative and clerical (*n* = 23), healthcare scientist (*n* = 4). Other respondent roles were students (*n* = 10), scientific and technical (*n* = 3).

Educational and ‘advising’ purposes were also prominent among three occupational roles:

- Medical and dental respondents (46.7% chose ‘teaching or presentations’, 42.2% chose ‘sharing information with, or advising, other staff and colleagues’.
- Nursing and midwifery respondents (34% chose ‘teaching or presentations’, 51.1% chose ‘sharing information with, or advising, other staff and colleagues’.
- Administrative and clerical respondents (52.2% chose ‘sharing information with, or advising, other staff and colleagues’).

### Data mining – Interpretation of questions

There was some overlap between teaching or presentations and sharing information with, or advising, other staff or colleagues, but generally these are distinct uses. Of the 52 respondents who selected ‘teaching or presentations’, 37 (71%) also selected ‘sharing information with, or advising other staff and colleagues’. Of the 86 respondents selecting ‘sharing information with, or advising, other staff and colleagues, 37 (43%) also selected “teaching or presentations’.

The ‘research’ use in Question 2 appears to be associated with personal research by students (7/10 students chose ‘research’). In contrast, for nursing and midwifery roles 36.2% of respondents, chose ‘research’, as did 46.7% of those in medical and dental roles. Of the 21 medical and dental respondents choosing research, 9 also selected publication as a use. This was a much higher proportion than for respondents in other roles that had selected research. Research may be personal, a work project or funded (to some extent). Of the administrative and clerical group (*n* = 23), 14 (60.9% of the group) chose ‘research’, but how research is being interpreted by this group is unclear. Nearly as many in the group chose ‘personal and professional development’ as a use. There are expected variations in interpretation, and deleting this item leads to the greatest improvement in internal reliability among all the items within Question 2, but may reduce usefulness.

The use for ‘patient information, advising or educating patients, clients or families’ was fairly evenly spread among direct patient care occupational groups: allied health respondents (32.1%), medical and dental respondents (35.7%), and nursing and midwifery respondents (28.6%). This pattern would be expected, as these groups are all involved in direct patient care that requires advising patients or families.

The associated personal impacts for the use for ‘patient information, advising or educating patients, clients or families’ were mostly ‘gain new knowledge’ (24/28, 86% respondents) followed by ‘gain new skills’ and ‘improve my confidence’ (both 20/28, 71% respondents). The main associated service impacts were ‘more informed decision making’ or ‘contributed to personal/professional development’ (both 22/28, 79% respondents). This pattern would be expected and illustrates that library users were aware of a gap in their knowledge or understanding and were able to help patients with more confidence after using library services.

There is a medium/moderate correlation (Pearson correlation coefficient *r* is 0.316) between ‘update skills’ and ‘gain new skills’, suggesting that these categories are meaningful to the respondents, and scale satisfactorily.

### Data mining – Patterns among uses, personal and service impacts

Fifty respondents had used the information, knowledge or skills gained for direct patient care. Of these most were in medical/dental roles (40%), followed by nursing and midwifery (30%), allied health professionals (26%) and additional clinical services (4%). This use was mostly associated with use for ‘personal and professional development’, followed by educational purposes (teaching/presentations and sharing information). The most frequent associated personal impact was ‘gain new knowledge’ (40/50, 80%). The most frequently associated service impacts were contributions to ‘personal/professional development’, ‘more informed decision making’ (41/50, 82%, ‘improved quality of patient care’ (40/50, 80%) and ‘service development and delivery’. This pattern of impacts is plausible. Results of a query that concerned direct patient care and personal learning by the respondent should be associated with gaining new knowledge, which could also lead to more informed decision making, followed by improved quality of care and then contribute to service development or delivery.

About as many respondents (*n* = 47) indicated they would probably use the information, knowledge or skills gained for direct patient care – the main occupational groups represented here were (in descending order) allied health professionals, nursing and midwifery, medicine and dental, students, and additional clinical services. The medicine and dental group were more likely to select ‘have used’ for direct patient care than ‘probably will use’ for direct patient care.

In the categories of use, there is ‘personal and professional development’, and one of the possible service impacts is ‘contributed to personal or professional development’ (immediately or in the future). We would expect a high number of respondents to tick both categories if they had selected either the use or the service impact categories. Of the 136 respondents who selected ‘personal and professional development’ among the use categories, 111 also selected ‘contributed to personal and professional development’ as a service impact. Looking at this in reverse, 140 respondents selected ‘had an immediate contribution’ in ‘contributed to personal and professional development’ in service impact, and of those 111 (79.3%) ticked ‘have used’, and 13 (9.3%) ticked ‘probably will use’ in the use categories. Overall, there is around an 85%–90% overlap which indicates consistency of interpretation and response.

Examining in detail the responses among different occupational groups for service impacts, the occupational groups with direct patient care responsibilities frequently noted: contributed to personal/professional development, more informed decision making, improved the quality of patient care/facilitated collaborative decision making/service development and delivery (Table 2).

**TABLE 2:** Service impacts (immediate and future) by selected occupational groups

Contributed to…	Allied health professionals (*n* = 48)	Medicine and dental (*n* = 45)	Nursing and midwifery (*n* = 47)
Personal and/or professional development	38 (*5*)	32 (*7*)	36 (*6*)
More informed decision making	19 (*17*)	22 (*11*)	20 (*12*)
Improved the quality of patient care	6 (*26*)	11 (*20*)	10 (*19*)
Facilitated collaborative decision making	5 (*9*)	9 (*12*)	8 (*9*)
Service development and delivery	9 (*21*)	11 (*15*)	6 (*20*)

*Note*: Italicised value in brackets represents probable future impact.

The patterns among the occupational groups are similar. The most frequent immediate service impact was personal and/or professional development (for 79.2% allied health professionals, 71.1% medicine and dental, 76.6% nursing and midwifery). More informed decision making was second for all groups, with the medicine and dental, nursing and midwifery groups more likely to report an immediate rather than a future impact whereas for allied health professionals there was little difference (39.6% immediate, 35.4% probable future).

Thinking of situations where people might want to ‘confirm prior knowledge or refresh your memory’, these might feasibly include for direct patient care or for teaching/presentation purposes. Of the 102 respondents that chose this personal impact, the information had been used for direct patient care by 25 (25%), teaching or presentations by 28 (27%) and for research by 43 (42%). As indicated earlier, research may cover quite a broad spectrum of activities, from long‐term personal research interests or funded projects through to one‐off student assignments.

Relatively few respondents (14/187, 8%) chose publication as a use of the information provided via the library services. Most of the respondents also selected personal and professional development (as another use, and as a service impact). Nine (5%) also indicated a research use.

Library managers might wish to identify instances of possible cost savings. One of the personal impacts is ‘save my time’, which potentially could be linked to cost savings. There is, however, no specific associated pattern with ‘save my time’. Of the 94 respondents who ticked the use category ‘save my time’, 78 (83%) also indicated personal and professional development as a use, 75 (80%) also indicated ‘sharing information with, or advising other staff’ and 70 (75%) indicated ‘research’ as uses. The main immediate contribution to service impacts was ‘more informed decision making’ – which suggests that time was saved by deciding more quickly on a course of action. The probable future contributions to service impact were, in descending order ‘improved the quality of care’, ‘contributed to services development or delivery’, and ‘saved money or contributed to financial effectiveness’. The latter category suggests that respondents are aware of the costs of their time.

Another impact, for service impact, that is associated with reducing costs associated with bad decisions, is ‘reduced risk or improved care’. Of the 69 respondents selecting this, most indicated ‘probable future contribution’ (*n* = 55, 80%) and only 14 indicated that the information provided had an immediate contribution. Most of the latter 14 respondents were using the library for personal and professional development.

### Data mining – Immediate and probable future personal and service impacts

Examining the patterns of responses for ‘gain new knowledge’, the pattern of immediate and probable future contributions to service impact are plausible. The recognition of an immediate contribution is easier for:

- contributed to personal or professional development (76.2%);
- more informed decision making (40.9%).

whereas, for the following outcomes, where other people might be involved, plus time and further work, immediate contributions are less than probable future contribution:

- reduced risk and improved safety (immediate 7.9% vs. 31.1% probable future);
- improved the quality of care (immediate 17.1% vs. 42.7% probable future);
- saved money or contributed to financial effectiveness (immediate 1.8% vs. 31.1% probable future);
- contributed to services development or delivery (immediate 18.9% vs. 45.1% probable future);
- facilitated collaborative working (immediate 15.9% vs. 26.2% probable future).

Interestingly, perhaps, there was less difference in the frequency of immediate and future service impacts for ‘facilitated collaborative decision making’, but the numbers are relatively small – around 20% for each occupational group (see Table 2).

The patterns of service impacts associated with ‘improve my confidence’, ‘gain new skills’, and ‘update new skills’ personal impacts are overwhelmingly concerned with immediate contributions to personal and professional development (87.1%, 83.8%, and 83.2% respectively). There is a wide scatter of responses for probable future service impacts, with the most frequently cited being ‘improved the quality of care’, and ‘contributed to services development or delivery’. As those personal impacts are likely to be associated frequently with information literacy training, it is good to know that respondents can clearly visualise how they might apply such training to more efficient and effective working in practice.

A similar pattern of service impacts is associated with ‘generate new ideas’ as a personal impact. The immediate contribution to service impact is ‘contributed to personal or professional development’, and the probable future service impacts are principally ‘improved the quality of care’, ‘more informed decision making’, and ‘contributed to services development or delivery’.

### Content validity

We interviewed 10 library users regarding their understanding of the content of the questionnaire. This included two administrative and clerical staff, one nurse, one medicine and dental, two allied health professionals, one health care scientist, one scientific and technical and two students, representing all the professional groupings on the questionnaire.

On the whole, respondents found the questionnaire understandable. For section two (uses of Information), a couple of respondents did not understand the terms commissioning or contracting, but in section three (personal impacts), four (service impacts) and five (professional roles), there were no problems. Section 1 (reasons for using the library service) were the most problematic. Although terms for such common or self‐explanatory library services such as literature search, supply of an article, training, access to information, study space or IT facilities were understood, no respondent understood ‘current awareness or alert service’, most did not understand the term ‘clinical or outreach librarian’, and a couple of respondents did not understand the term ‘journal club’.

## DISCUSSION

We set out to conduct additional validation of a short generic questionnaire to measure the impact of health library services. Face validity was established in an earlier version of the questionnaire (Ayre et al., 2018). This study which demonstrates that the questionnaire has reliability (internal consistency) and content validity shows that this questionnaire is now suitable for routine use. Performing this additional testing is novel in this field, as no other questionnaires evaluating the quality or impact of health library services have reported going beyond initial validation, such as face validity or piloting. Several researchers based at McGill University, Canada have worked for many years on development and validation of the Information Assessment Method (IAM) (Granikov et al., 2020), which was originally aimed at electronic information for health professionals but now comes with variations for different types of electronic information provision (Bujold et al., 2018, Pluye et al., 2015 for parenting information). For health library impact studies, the usual pattern has been to incorporate the findings of large mixed methods studies to help improve the content of the questions and methodology. For example, Urquhart and Hepworth (1995) used the categories of clinical decision making derived from the NLM study of MEDLINE searching (Lindberg et al., 1993) together with questions from the Rochester impact study (Marshall, 1995), and Dunn et al. (2009) reviewed and revised the procedures and research instruments for the second US (Rochester) impact study (Marshall et al., 2013).

It is encouraging that library users generally found the items on the questionnaire understandable. However, it is of concern that users do not understand the terms that libraries are using to describe their services. Particular misunderstandings included current awareness or alert services, clinical and outreach librarians and journal clubs; these are services where libraries are more proactive and likely to provide added value. As well as potentially downplaying the potential impact of the library service, this may cause practical issues in terms of non‐completion of the questionnaire because respondents may not realise the questionnaire or service applies to them, or else they may not understand the relationship to ‘one recent issue of library use’. This use of the critical incident technique (as described in Ayre et al., 2018) is an important feature of the questionnaire, otherwise libraries will only receive a general impression of satisfaction, rather than an understanding of particular impacts. Impacts are more meaningful if they can be related to one incident of library use, as library users can recall the details around the incident of library use, and the type of associated personal and service impacts (Urquhart et al., 2003). Interview respondents confirmed they did understand this, but they were not asked to describe their actual single use of the services, which would have provided an additional understanding of this factor. Going forward, to improve responses, libraries may need to adapt their terminology or send the questionnaire out with a reminder of the service supplied. Providing more contextual information may improve the responses obtained even for those services which users appeared to be able to recall.

There has been considerable research on the presentation order effects on document relevance judgements by database users (Eisenberg & Barry, 1988), although the advent of search engine searching such as Google probably means that library service users expect that the most relevant documents come first. This suggests that the order of categories in question one should reflect the likely frequency of single library service use to be investigated. If current awareness service/alerts is a major component of the library service offering, then this category should come near the top. If journal club services are much less prominent, then this category should come nearer the bottom. This will be further investigated with library practitioners within English LKS. In the meantime, the guidance for use of the questionnaire (https://kfh.libraryservices.nhs.uk/value-and-impact-toolkit/) will be modified regarding the provision of contextual information but the questionnaire will remain the same (Appendix 2).

In terms of reliability, examination of the patterns of responses, and the Cronbach alpha calculation indicated the questionnaire (Question 2 onwards) is understood by the respondents, and the responses are consistent and reliable. One easily demonstrated example is the overlap between the responses for the ‘use’ (purpose) of personal and/or professional development and the service impact of personal and/or professional development. Lack of overlap would indicate problems in interpretation. Those doing impact analyses need to be alert to the variation in meanings attached to ‘research’.

Patterns of contributions to service impact – whether immediate or probable future contributions are plausible, as the service impacts that might require collaboration, or further time and work are more likely to be probable future impacts. A questionnaire for a relatively narrow and cohesive user group (such as those studied for IAM impact surveys, Granikov et al., 2020) may be easier to validate, when there is only one information source being studied, and impacts more likely to be immediate. Library services are more varied, as already noted in the discussion on order of categories for question one, and have a major role in supporting education and continuing professional development.

One limitation of the validation study is that most of the responses were concerned with only two of the possible library services that could be included: literature or evidence searches, and training or e‐learning. This is partly attributable to the data set used for the validation study or the confusion with library service terminology, rather than a lack of use of the wider range of library services. Another data set supplied by a different group of libraries (with shortened version of the impact survey) indicated that around 10% of the uses were for ‘access to electronic or print information’ and around 25% for ‘supply of an article, book or document’. Questionnaire distribution may be easier for more personal services offered by libraries rather than access to digital material or electronic resources. The advantages of the focus on personal services is that (1) they are demanding of staff time and therefore require justification of impact; and (2) the exact library service use should be easy to recall for the respondent. On the other hand, not assessing the full impact of information obtained from digital resources means that the impact of investment in journal resources and the impact of information provided for the online (only) users of library services may be neglected.

Administration of the impact questionnaire should not add to routine staff workload, but it is important to obtain a good response rate. Internet surveys are easy to administer and can achieve a wide coverage of the population to be surveyed, but the difficulty is the low response rate – which can typically range from 6% to 15% (Evans & Mathur, 2018). A more personalised approach, such as sending the impact questionnaire with the results of a literature search, or document supplied to the individual user, is more likely to gain a response (and reminders are easier to manage). Weightman et al. (2009) provide some general evidence‐based advice for impact survey design and procedures.

Although the questionnaire was aimed at use within health libraries serving the English NHS, the questionnaire could be used more widely. Use of the questionnaire across England will enable the creation of a national impact data set for health library services. It is likely that changes in health and library services will require monitoring of the content validity of the questionnaire. There is international debate around the need, how and what to measure in relation to impact (Hughes et al., 2019; Ibragimova & Korjonen, 2019; Urquhart & Turner, 2016) but previous national library studies elsewhere are restricted to specific impacts such as direct patient care or on accreditation and have not reported their reliability and validity (Marshall et al., 2013; Marshall et al., 2014; Ritchie et al., 2020). Confirming the validity and reliability of the questionnaire elsewhere would facilitate the development of an international evidence base for health libraries for use in advocacy and planning.

## CONCLUSION

The generic impact questionnaire is a reliable and valid tool for demonstrating the impact of Library and Knowledge Services in England. Libraries who use the questionnaire should think carefully regarding who and when to target when using the questionnaire and ensure that contextual information about the library services being assessed is provided as necessary. The questionnaire has potential for wider use across the UK and internationally, but if used in other contexts, would benefit from additional validity checks to ensure terminology is transferable.

## CONFLICT OF INTEREST

The authors declare no potential conflict of interest.

## AUTHOR CONTRIBUTIONS

Christine Urquhart and Alison Brettle conceived the study. Christine Urquhart was responsible for the quantitative methods and Alison Brettle for the qualitative methods. Alison Brettle secured the funding, ethics and managed the project. Christine Urquhart and Alison Brettle contributed to the final version of the manuscript.

## APPENDIX A QUESTIONNAIRE

### Impact of library services questionnaire

**Table:**

Impact of library services		
This short survey is to collect information about the value of library services. The questions were developed by a Value and Impact task and finish group working for NHS libraries in England (part of the NHS Knowledge for Healthcare framework) *and validated by the University of Salford*. The data you provide will help us understand and demonstrate the contribution of library services. *Version 2.0 June 2020*.		
**1. You recently used the library service for:**		
Current awareness or alerts	□	
Literature search or evidence search	□	
Supply of an article, book or document	□	
Training or e‐learning	□	
Access to electronic or print information	□	
Clinical or outreach librarian service	□	
Study space	□	
IT facilities	□	
Journal club	□	
**2. From that single use of library services or resources how did you use, or how might you use, the information, knowledge or skills gained? (Tick any that apply)**		
	Have used	Probably will use
Personal or professional development	□	□
Direct patient care	□	□
Teaching or presentations	□	□
Sharing information with, or advising, other staff or colleagues	□	□
Patient information, advising or educating patients, clients or families	□	□
Developing guidelines/guidance/pathways/policies	□	□
Audit	□	□
Research	□	□
Organisational/service development/business planning	□	□
Legal or ethical questions	□	□
Commissioning or contracting	□	□
Publication	□	□
None of the above	□	□
**3. From that single use of library services or resources how did the information, knowledge or skills gained help? (Tick any that apply)**		
Confirm prior knowledge or refresh my memory	□	
Gain new knowledge	□	
Generate new ideas	□	
Update skills	□	
Gain new skills	□	
Improve my confidence	□	
Save my time	□	
None of the above	□	
**4. Did your use of library resources or services contribute to any of the following impacts? (Tick any that apply)**		
	Had an immediate contribution	Probable future contribution
Reduced risk or improved safety	□	□
Improved the quality of patient care	□	□
Saved money or contributed to financial effectiveness	□	□
More informed decision making	□	□
Contributed to service development or delivery	□	□
Facilitated collaborative working	□	□
Contributed to personal or professional development	□	□
None of the above	□	□
**5. What is your main role?**		
If it is unclear which option your role fits into you can check the guidance (right click on the link and open in a new window or check online) http://content.digital.nhs.uk/media/11198/Appendix‐A‐Staff‐Group‐Definitions‐v40/pdf/Appendix_A_Staff_Group_Definitions_v4.0_Final.pdf		
Additional Clinical Services	□	
Administrative & Clerical	□	
Allied Health Professionals	□	
Estates & Ancillary	□	
Healthcare Scientists	□	
Medicine & Dental	□	
Nursing & Midwifery	□	
Scientific & Technical	□	
Students	□	

## APPENDIX B REVISED TEXT FOR KfH WEBSITE

### B.1

http://kfh.libraryservices.nhs.uk/value-and-impact-toolkit/kfh-impact-tools/generic-questionnaire/

*Revisions in italics*

It is envisaged the questionnaire is used in two ways:

1. As a generic survey of customers which might be sent to all customers/a subgroup of customers on a regular basis (e.g. annually).
2. As a targeted survey of the impact of a specific service (e.g. a literature search or information skills training).

The questionnaire has been kept deliberately generic to be applicable to a wide range of situations and uses *and has been validated for this purpose*.

*The validation demonstrated that the questionnaire was reliable. Any local changes to core questions (Sections 2–4) could reduce the reliability of the questionnaire. Any changes would also mean that we cannot combine data across LKS. Additional questions can be added to these core questions for local use*. For example, you may also wish to request name and e‐mail address if you plan to follow up with case studies or interview requests.

*The validation highlighted confusion in terminology of library services offered (Section 1). The most common services have been offered first. Services not provided by a particular LKS could be removed from this section*.

If LKS are seeking feedback for a specific instance, a covering e‐mail specifying the incident about which feedback *is sought is recommended*. (e.g. We recently provided you with a literature search on XXX… we would be interested to know how you used the resulting evidence.)

The questionnaire was developed by the Value and Impact Task and Finish Group from the Quality Work Stream of the Knowledge for Healthcare programme, has been piloted on a range of library services and *validated by the University of Salford*.
